# The Rice AAA-ATPase OsFIGNL1 Is Essential for Male Meiosis

**DOI:** 10.3389/fpls.2017.01639

**Published:** 2017-09-27

**Authors:** Peipei Zhang, Yingxin Zhang, Lianping Sun, Sittipun Sinumporn, Zhengfu Yang, Bin Sun, Dandan Xuan, Zihe Li, Ping Yu, Weixun Wu, Kejian Wang, Liyong Cao, Shihua Cheng

**Affiliations:** ^1^Key Laboratory for Zhejiang Super Rice Research and State Key Laboratory of Rice Biology, China National Rice Research Institute, Hangzhou, China; ^2^National Key Laboratory of Crop Genetic Improvement, Huazhong Agricultural University, Wuhan, China

**Keywords:** *Oryza sativa*, meiosis, male sterility, *OsFIGNL1*, chromosomes

## Abstract

Meiosis is crucial in reproduction of plants and ensuring genetic diversity. Although several genes involved in homologous recombination and DNA repair have been reported, their functions in rice (*Oryza sativa*) male meiosis remain poorly understood. Here, we isolated and characterized the rice *OsFIGNL1* (*OsFidgetin-like 1*) gene, encoding a conserved AAA-ATPase, and explored its function and importance in male meiosis and pollen formation. The rice *Osfignl1* mutant exhibited normal vegetative growth, but failed to produce seeds and displayed pollen abortion phenotype. Phenotypic comparisons between the wild-type and *Osfignl1* mutant demonstrated that OsFIGNL1 is required for anther development, and that the recessive mutation of this gene causes male sterility in rice. Complementation and CRISPR/Cas9 experiments demonstrated that wild-type *OsFIGNL1* is responsible for the male sterility phenotype. Subcellular localization showed that OsFIGNL1-green fluorescent protein was exclusively localized in the nucleus of rice protoplasts. Male meiosis in the *Osfignl1* mutant exhibited abnormal chromosome behavior, including chromosome bridges and multivalent chromosomes at diakinesis, lagging chromosomes, and chromosome fragments during meiosis. Yeast two-hybrid assays demonstrated OsFIGNL1 could interact with RAD51A1, RAD51A2, DMC1A, DMC1B, and these physical interactions were further confirmed by BiFC assay. Taken together, our results suggest that *OsFIGNL1* plays an important role in regulation of male meiosis and anther development.

## Introduction

Male reproductive development is a complex biological process, an understanding of which can lend insight for breeders into traits that increase yield and ensure rice reproductive ability. Pollen fertility also is a key factor in rice grain yield (Zhang et al., 2010; Zhou et al., 2011). During male reproductive development, the anther wall consists of the epidermis, the endothecium, the middle layer and the tapetum (Zhang et al., 2011). The morphology of four somatic layers of the anther wall undergoes dynamic changes, along with the haploid male gametophyte (pollen) generated from diploid pollen mother cells (PMCs) (Tao et al., 2007; Zhang et al., 2011). The formation of pollen grains in plants includes PMC formation, male meiosis, two mitotic processes of microspore and starch filling, and maturation of pollen grains (McCormick, 2004). Meiosis is a critical step in gametogenesis, during which two rounds of cell division follow a single round of DNA replication, ending with formation of four haploid cells. It is known that meiosis plays a central role in stabilization of genomic information and establishment of genetic diversity (Nonomura et al., 2004). During meiosis, several key events, including synaptonemal complex assembly, homologous chromosome pairing, and chiasma formation, could coordinately assure accurate segregation of meiotic chromosomes (Li and Ma, 2006; Shen et al., 2012; Luo et al., 2013). Disturbing any step of this progress leads to severe dysfunction of meiotic cells, including aberrant chromosomes, abnormal pairing of homologs, reduction of chiasma frequency, and infertility.

In eukaryotes, meiotic recombination starts from meiotic prophase I, during which a conserved protein SPO11 generates double-strand breaks (DSBs) and covalently links to the 5′ends of DNA at the incision sites of the DSBs (Keeney et al., 1997). In yeast, the covalently linked SPO11 are subsequently removed by the Mre11/Rad50/Xrs2-Nbs1 (MRX/N) complex and Sae2/Com to form 3′-single-stranded tails (Neale et al., 2005; Filippo et al., 2008; Milman et al., 2009; Edlinger and Schlogelhofer, 2011). In the following step, RAD51, together with the meiosis-specific DMC1, bind to single-stranded DNA generated from a DSB, then search and attach to a homologous template to form the double Holliday junction (dHJ) (Cloud et al., 2012). And then, meiotic DSBs are finally repaired to produce crossovers (COs) and non-crossovers (NCOs). In *Arabidopsis*, *AtRAD51, AtDMC1, AtRAD51C*, and *AtXRCC3* have been shown to be important for homologous recombination and DNA repair in plant meiosis (Pradillo et al., 2014). In *Arabidopsis thaliana*, *mnd1* and *hop2* mutants have been characterized that exhibit chromosome fragmentation and sterility associated with DNA repair during meiosis (Uanschou et al., 2013). In eukaryotes, Hop2-Mnd1 heterodimerizes and stimulates the DNA strand exchange activity of RAD51 and DMC1 (Pezza et al., 2010; Bugreev et al., 2014). In another study, the *Arabidopsis* AtBRCA2 was reported to be essential for DNA repair (Seeliger et al., 2012). AtBRCA2 is another key recombination co-factor that interacts with AtRAD51 and AtDMC1, and mediates the recruitment of AtRAD51 and AtDMC1 during meiotic recombination (Dray et al., 2006; Seeliger et al., 2012). The orthologs of these genes play conserved roles in rice. For example, two RecA homologs, the RAD51 and DMC1 proteins, also have been shown to catalyze strand exchange between homologous chromosomes (Rajanikant et al., 2008; Sakane et al., 2008; Morozumi et al., 2013). In rice, a recent study revealed that OsMRE11 is essential for homologous synapsis and DSB ends processing (Ji et al., 2013). Additionally, OsSDS are known to function in DNA DSB formation during rice meiosis (Wu et al., 2015). Mutations in these genes cause abnormalities in meiosis, including chromosome fragmentation, defective homologous pairing and synapsis, and eventually lead to severe defects in male and female fertility in rice (Couteau et al., 1999; Bleuyard and White, 2004; Li et al., 2004, 2005; Pradillo et al., 2014). However, many aspects of the process of meiosis remain poorly understood in plants.

FIDGETIN is a member of the AAA (ATPase Associated with diverse cellular Activities) protein superfamily, the mutation of which causes multiple developmental defects in mice. Fidget mice exhibit cell cycle delay, insufficient growth of the retinal neural epithelium, side-to-side head shaking and circling, and small eyes (Cox, 2000). FIDGETIN, as well as the closely related FIDGETIN-LIKE 1 (FIGNL1) and FIDGETIN-LIKE 2 (FIGNL2) proteins also were identified in mouse (Cox, 2000; Yang et al., 2005). FIGNL1 is a homolog protein of FIDGETIN (Cox, 2000), and reportedly plays a critical roles in meiosis (Girard et al., 2015). Previous studies have demonstrated that FIGNL1 plays an important role in animal meiotic cell division. In male mice, *FIGNL1* is most highly expressed during the spermatocyte stage (L’Hote et al., 2011). Recently, it was reported that FIGNL1 specifically interacts with SMO-1 in *Caenorhabditis elegans*, with mutation of *FIGNL1* resulting in defective gonad formation and the sterile phenotype (Luke-Glaser et al., 2007; Onitake et al., 2012). In *Arabidopsis*, the FIGNL1 was found to suppress formation of genome-wide meiotic COs, possibly by hindering the strand exchange activity of two conserved recombinases AtDMC1 and AtRAD51 (Girard et al., 2015). Despite advances in understanding the meiotic process, the potential molecular mechanism and function of FIGNL1 is still far from clear.

Considerable research has been conducted to elucidate the mechanisms underlying meiosis, particularly during male gametogenesis in plants. However, the functions of many genes involved in the meiotic process remain unknown. In this study, using a map-based cloning strategy, we isolated and functionally characterized a rice AAA-ATPase gene *OsFIGNL1. Osfignl1* male meiocytes exhibited abnormal chromosomes during meiosis, which finally resulted in aborted pollen grains and entire male sterility of the plants. This work sheds new light on OsFIGNL1 regulation of meiotic chromosome behavior during male gametogenesis in monocot plants.

## Materials and Methods

### Plant Materials, Growth Conditions, and Mutant Phenotyping

The wild-type used in this study was *Oryza sativa* spp. *indica* cv. Zhonghui 8015 (Zh8015). Mutagenesis of Zh8015 was performed by treatment with ^60^Co-γ ray radiation, resulting in identification of the complete male sterility mutant *Osfignl1.* The *Osfignl1* mutant, as the pollen acceptor, was crossed with wild-type and Zhonghua11 (*japonica*), respectively. The heterozygous F_1_ plants were then self-pollinated to generate a BC_1_F_2_, and an F_2_ population for genetic analysis and mapping of the target locus. All plants were grown in paddy fields in Lingshui, Hainan Province, and Hangzhou, Zhejiang Province, China. Planting density and crop management followed commercial rice production practices. The mutant exhibited genetic stability in both Hainan and Zhejiang.

To assess the viability of mature pollen grains, anthers were collected from the wild-type and *Osfignl1* mutant at anthesis stage and stained with 1.2% iodine-potassium iodide solution (I_2_-KI) to observe starch accumulation in pollen grains by light microscopy (Li et al., 2010). The wild-type and mutant florets at different developmental stages were fixed in FAA solution (formalin:acetic acid:50% ethanol were 5:5:90, by volume) to observe developmental progression of embryo sacs as described previously (Wang C.L. et al., 2016). The ovaries were finally captured using a laser confocal scanning microscope (Zeiss, Germany).

### Map-Based Cloning of the *OsFIGNL1* Gene and Complementation of the *Osfignl1* Mutant

The F_2_ mapping population was generated from a cross between the *Osfignl1* mutant and *O. sativa* spp. *japonica* cv. Zhonghua11. Two thousand and three hundred plants of the F_2_ population showing the male sterility phenotype were selected for gene mapping. DNA was extracted from fresh leaves using the modified CTAB method (Murray and Thompson, 1980). The InDel markers were designed based on polymorphisms between *japonica* Nipponbare and *indica* 9311 (Shen et al., 2004). The InDel marker sequences are listed in Supplementary Table S1.

For functional complementation, an 11.9 kb genomic DNA amplicon containing the entire *OsFIGNL1* coding region, 2.2-kb upstream and 1.0-kb downstream sequence was obtained by PCR amplification using primer pairs COM-*OsFIGNL1*. The PCR product was inserted into the binary vector pCAMBIA1300^1^ to generate *pCAMBIA1300-OsFIGNL1* construct using an *Eco*RI enzyme site. The *pCAMBIA1300-OsFIGNL* binary plasmids were introduced into the *Agrobacterium tumefaciens* strain EHA105 and transformed into calli induced from young panicles of *Osfignl1*. The transgenic plants were further identified by PCR amplification using the primer COM-JD (Supplementary Table S1). To confirm whether the fertility of *Osfignl1* mutant was restored by *OsFIGNL1* gene, the pollen grains of T_0_ transgenic plants were stained with 1.2% I_2_-KI and observed under a light microscope.

To generate the Cas9 targeting vector, we used the pCAS9-*Aar*I vector containing a codon-optimized Cas9 driven by a maize ubiquitin promoter, the *OsU3* promoter and sgRNA scaffolds, as well as the Cas9 expression backbone vector (Li et al., 2016), with *Aar*I restriction enzyme (Fermentas). The targeting sequence primer pairs Cas*-OsFIGNL1* are listed in Supplementary Table S1. The *OsU3* promoter was used to drive expression of the sgRNA. The annealed gRNA oligonucleotide pair was cloned into pCAS9-*Aar*I binary vectors and introduced into the *Agrobacterium* strain EHA105. Transformed calli were induced from Zh8015 seeds for *Agrobacterium*-mediated transformation as previously described (Lin et al., 2009). The T_0_ transgenic mutant plants were confirmed by PCR using primer Cr-JD (see Supplementary Table S1).

### RNA Isolation and qRT-PCR Analysis

Total RNA was isolated from roots, stems, leaves, and anthers at different developmental stages using the RNAeasy Mini Kit (Tiangen, China), as per manufacturer’s instructions. Total RNA was used for reverse transcription (RT) with the ReverTra Ace quantitative PCR RT Master Mix Kit with gDNA Remover (Toyobo, Japan). qRT-PCR was performed with a LightCycler 480 (Roche, Germany) using SYBR Premix Ex Taq II (Takara, Japan) according to manufacturer’s instructions. The reaction program was run as follows: 94°C for 4 min initial denaturation, then 94°C for 15 s, 58°C for 30 s, and 72°C for 15 s for 45 cycles. The primer pairs S12 were designed to examine the expression of *OsFIGNL1* in the wild-type and *Osfignl1* mutant. The rice *OsActin1* gene was chose as a control to normalize expression data. Each reaction was performed with three replicates. All primers are listed in Supplementary Table S1.

### Resin Sections of Wild-Type and Mutant Anthers

Thin sections of developing anthers were performed on standard plastic sections according to a previously described method (Li et al., 2006). Spikelets at various development stages were collected and fixed in FAA solution overnight at room temperature. After dehydration in a graded ethanol series from 50 to 100%, the samples were embedded in Technovit 7100 resin (Heraeus, Kulzer, Germany), and polymerized at 45°C. Transverse sections of 2 μm were cut using a Leica RM2265 Fully Automated Rotary Microtome, stained with 0.25% toluidine blue (Chroma Gesellshaft Shaud), and photographed using a Leica DM2000 light microscope.

### Transmission Electron Microscopy (TEM) and Scanning Electron Microscopy (SEM)

For TEM observation, spikelets at different developmental stages were fixed with 3% (w/v) paraformaldehyde and 0.25% glutaraldehyde in 0.2 M sodium phosphate buffer (pH 7.2) overnight at 4°C, then rinsed twice using 0.1 M phosphate buffer (pH 7.0). Samples were then post-fixed in 2% osmium tetroxide in PBS, pH 7.2. The samples were dehydrated using an ethanol series from 30 to 100%, then embedded in acrylic resin. Ultra-thin sections were cut and collected on uncoated nickel grids, and double stained with 2% uranyl acetate and 2.6% lead citrate aqueous solution. The pictures were examined with a Hitachi H-7650 transmission electron microscope at 80 kV.

For SEM observation, mature anthers of the wild-type and the mutant were pre-fixed in 0.1 M sodium phosphate buffer containing 2.5% glutaraldehyde (pH 7.0), overnight at 4°C, then rinsed twice using 0.1 M phosphate buffer (pH 7.0). The samples were rinsed with the same buffer and post-fixed for 1.5 h in 2% OsO_4_ in PBS, pH 7.2. Following ethanol dehydration and then exchanged three times with isoamyl acetate. The fixed samples were processed for critical point drying using liquid CO_2_, and gold coated. The samples were observed using a scanning electron microscope (TM-1000 Hitachi) with an acceleration voltage of 10 or 15 kV.

### Meiotic Chromosome Preparation

Young panicles (80–90 mm), including both wild-type and *Osfignl1* spikelets in meiosis were fixed with 3:1 ethanol:acetic acid (EAA), and stored at 4°C until observation. Microsporocytes undergoing meiotic division were squashed on glass slides and stained with an acetocarmine solution. Slides with chromosomes were frozen in liquid nitrogen. After removing the coverslips, the slides were dehydrated through an ethanol series (70, 90, and 100%). Male meiotic chromosomes were counterstained with 4′,6-diamidino-phenylindole (DAPI). Chromosome images were captured using Nikon Eclipse 90i microscope with Nikon Digital Camera DXM1200C and Nikon C-HGFI.

### Phylogenetic Analysis

The full-length amino acid sequence of OsFIGNL1 and protein sequence of its homologs from different species were retrieved through BLASTP search using the amino acid sequence of OsFIGNL1 from the NCBI database^2^. The multiple sequence alignment was created using Clustalw with the default parameters^3^. A phylogenetic tree was constructed with the neighbor-joining algorithm using MEGA 6.0 software (Tamura et al., 2013). Bootstrap values were calculated from 1000 bootstrap replicates.

### Subcellular Localization

To construct the subcellular localization plasmids, the full-length coding region of *OsFIGNL1* was amplified with the primer pair GFP*-OsFIGNL1* (Supplementary Table S1), with *Bam*HI and *Spe*I restriction sites added to the 5′ and 3′ ends, respectively, then ligated into the *pCAMBIA1305-GFP* expression vector (pCAMBIA^4^). Mesophyll protoplasts were isolated from 2-week-old Zh8015 plants. The empty vector control and recombinant construct plasmids (*pCAMBIA1305-OsFIGNL1-GFP*) were transformed into rice protoplasts and incubated for 48 h in the dark (Yoo et al., 2007). Fluorescence signal in the transformed protoplasts was imaged using a confocal laser scanning microscope (Zeiss, Germany). The *OsMADS3*-mCherry construct was used as nuclear marker (Wu et al., 2013).

### Yeast Two-Hybrid (Y2H) Assay and BiFC Assay

Full-length cDNA of *OsFIGNL1* transcripts were amplified and cloned into the Y2H bait vector pGBDT7, while cDNA of *RAD51A1*, *RAD51A2*, *DMC1A*, and *DMC1B* were amplified from young panicles and cloned into the Y2H prey vector, pGADT7 (Clontech). Plasmid inserts were confirmed by DNA sequencing. The two plasmid types were then co-transformed into the *Saccharomyces cerevisiae* Y2HGold strain according to the manufacturer’s instruction (Clontech). Transformants were grown on tryptophan-negative and leucine-negative synthetic dropout medium (SD/–Trp–Leu) then incubated on test plates lacking Leu, Trp, His, and Ade at 30°C for 4 days. For generation of BiFC vectors, cDNA of the rice *RAD51A1*, *RAD51A2*, *DMC1A*, and *DMC1B* were amplified from young panicles of *indica* Zh8015 and cloned at *Bam*HI-*Xho*I sites in *pSPYNE* to generate *pSPYNE-RAD51A1*, *pSPYNE-RAD51A2*, *pSPYNE-DMC1A*, and *pSPYNE-DMC1B* plasmids. The CDS of *OsFIGNL1* was cloned into *Bam*HI-*Xho*I sites of *pSPYCE*, resulting in *pSPYCE-OsFIGNL1* vector. The different combinations of constructs were transformed into the *Agrobacterium* train GV3101. All constructs were expressed under the control of the *35S* promoter. The procedure of BiFC was performed as described previously (Waadt et al., 2008). All primer pairs are listed in Supplementary Table S1.

## Results

### Morphological Characterization of the *Osfignl1* Mutant

**FIGURE 1:**
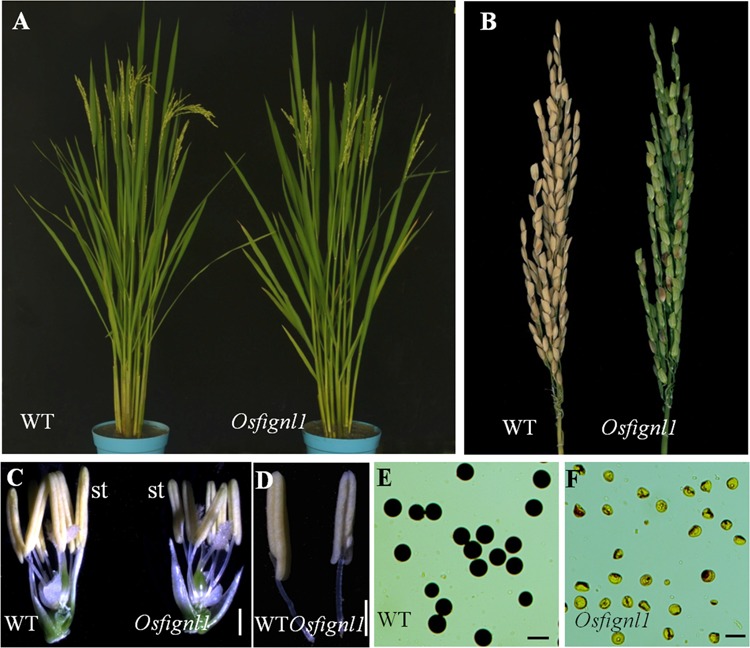
Phenotype comparisons between the wild-type (WT) and the *Osfignl1* mutant. **(A)** Comparison between a WT (left) and a *Osfignl1* mutant (right) plant after heading. **(B)** Comparison of a WT panicle (left) and a *Osfignl1* mutant panicle (right) at the harvest stage. **(C)** Comparison of a WT (left) and the *Osfignl1* mutant spikelet (right) after removal of palea and lemma. **(D)** Comparison of a WT (left) and a *Osfignl1* mutant anther (right). **(E)** I_2_-KI staining of WT pollen grains. **(F)** I_2_-KI staining of *Osfignl1* mutant pollen grains. st, stamen. Bars = 1 mm in **(C,D)**, 50 μm in **(E,F)**.

We obtained a complete male sterility mutant *Osfignl1* from an *indica* rice cultivar, Zhonghui 8015, induced by ^60^Co-γ ray radiation. Compared with the wild-type plants, *Osfignl1* displayed a normal phenotype during vegetative development, however, the mutant failed to set seeds (**Figures 1A,B**). Compared to wild-type anthers, the mutant anthers were relatively small, thin, and slightly yellow (**Figures 1C,D**). During the reproductive stage, mature pollen grains of wild-type anthers were darkly stained by 1.2% I_2_-KI, whereas the pollen grains of *Osfignl1* lacked starch and could not be stained (**Figures 1E,F**). To estimate female fertility of the *Osfignl1* mutant, we conducted reciprocal crosses between the wild-type and *Osfignl1* mutant. The mutant successfully set hybrid seeds when it was pollinated with wild-type pollen grains, indicating a normal megagametogenesis in the *Osfignl1* mutant (Supplementary Figure S1). To ascertain whether female organ development was influenced in the *Osfignl1* mutant, a total of 300 embryo sacs of the *Osfignl1* plants were examined for female fertility. Observation of normal female reproduction in *Osfignl1* was further supported by our cytological analysis of the *Osfignl1* embryo sac, which did not show any obviously abnormal morphology during embryo sac development (Supplementary Figure S2). We thus concluded that the sterility of spikelets in *Osfignl1* plants is caused by the disruption of male reproduction in rice. Genetic analyses showed that fertile plants and sterile plants followed an approximate 3:1 ratio for phenotypic segregation in F2 progenies, indicating that the sterility phenotype was caused by a single recessive mutation (fertile: sterile = 146: 42; χ^2^ = 0.57 < $\chi_{0.05,1}^{2}$ = 3.84).

### Abnormal Anther Development and Pollen Maturation of the *Osfignl1* Mutant

**FIGURE 2:**
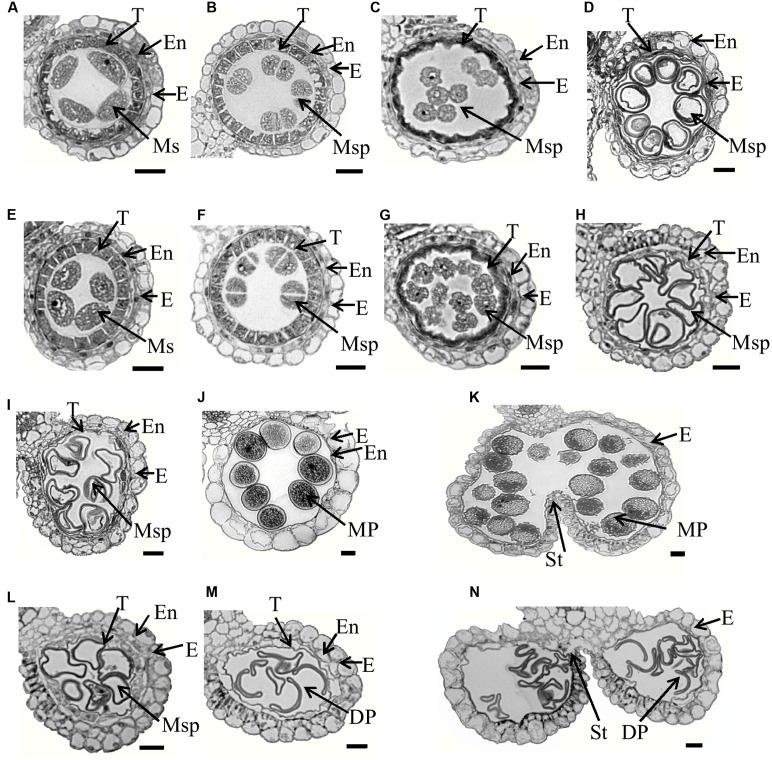
Semi-thin section comparison of anther development in the WT and the *Osfignl1* mutant. **(A–D)** and **(I–K)** show the WT; **(E–H)** and **(L–N)** show the *Osfignl1* mutant. **(A,E)** Meiosis stage. **(B,F)** Tetrads stage. **(C,G)** Young microspore stage. **(D,H)** Vacuolated pollen stage. **(I,L)** Bicellular pollen stage. **(J,M)** Mature pollen stage. **(K,N)** Anther dehiscence stage. E, epidermis; En, endothecium; DP, defective pollen; MP, mature pollen; T, tapetum; Ms, microsporocyte; Msp, microspore; St, stomium. Bars = 20 μm.

To characterize the defects in *Osfignl1*, we examined cross sections of anthers sampled from the wild-type and *Osfignl1* mutant spikelets of different developmental stages. No obvious differences in four somatic layers of the anther wall and microsporocytes were detected between the wild-type and *Osfignl1* mutant until the early microspore uninucleate stage (**Figures 2A–C,E–G**). PMCs of both the wild-type and the *Osfignl1* mutant appeared to undergo meiosis and form tetrads. The tapetal cells became vacuolated and deeply stained, and the middle layer was hardly visible and degenerated at the tetrad stage (**Figures 2B,F**). At the young microspore stage, microspores were released from the tetrads, while the tapetum became condensed, less vacuolated, and degenerated normally in the *Osfignl1* mutant, just like in wild-type anthers (**Figures 2C,G**). At the vacuolated pollen and bicellular pollen stages, the wild-type tapetum became more degenerated, the endothecium developed normally, and round-shaped, vacuolated microspores were formed in the anther locule. In contrast, the *Osfignl1* tapetum was slightly expanded, and microspores formed with an abnormal shape and no vacuoles (**Figures 2D,H,I,L**). At the mature pollen stage, mature wild-type pollen grains filled with starch granules were densely stained, and the tapetum completely disappeared. However, at this stage in the *Osfignl1* anther, the cells of the endothecium appeared expanded, the tapetum remnants abnormally persisted with no cell contents, and microspores degenerated with an irregular shrunken shape (**Figures 2J,M**). At the anther dehiscence stage, when the filament elongated, the endothecium in wild-type anthers was almost completely degraded and invisible. At the same stage, the *Osfignl1* tapetum had not yet disappeared completely and the endothecium degenerated further (**Figures 2K,N**). Two adjacent pollen sacs of the wild-type were able to merge into a single locule. In contrast, pollen sacs in *Osfignl1* were not linked together and aborted pollen grains were released from the anther through individual open stomium, showing that anther dehiscence also was affected in *Osfignl1* (**Figures 2K,N**). These observations together indicate that defects in microspore formation and anther development occur in the *Osfignl1* mutant.

**FIGURE 3:**
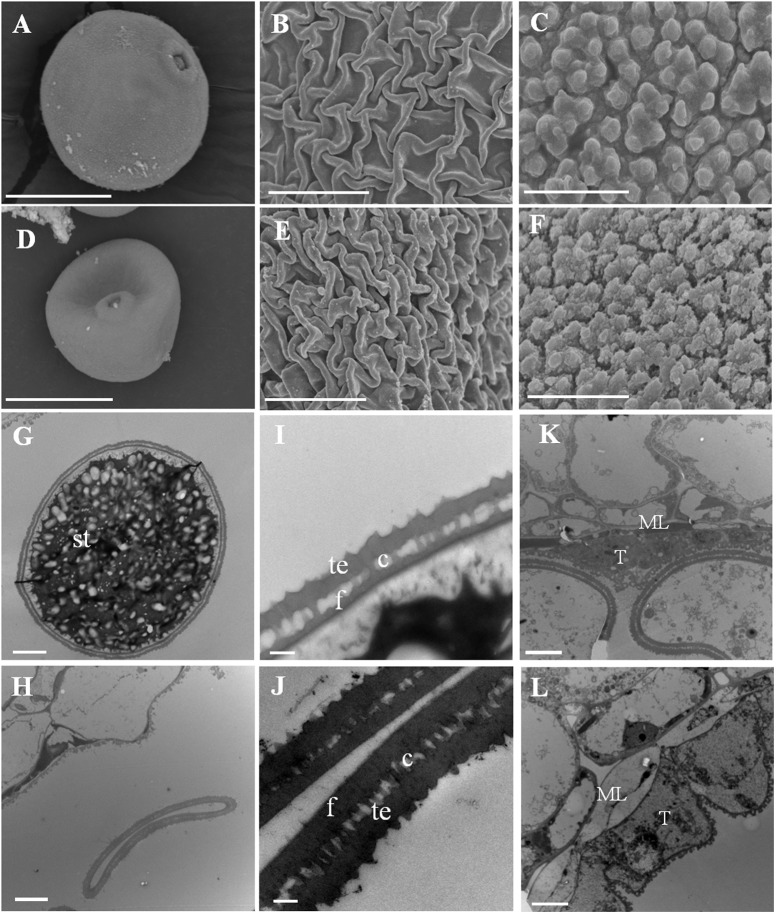
Scanning electron microscopy (SEM) and transmission electron microscopy (TEM) observation for WT and *Osfignl1* mutant anther. **(A)** Scanning electron microscopy image of the mature WT pollen grain. **(B)** Scanning electron microscopy image of the WT anther epidermis. **(C)** Scanning electron microscopy image of the WT pollen exine. **(D)** Scanning electron microscopy image of the *Osfignl1* pollen. **(E)** Scanning electron microscopy image of the *Osfignl1* anther epidermis. **(F)** Scanning electron microscopy image of the *Osfignl1* pollen exine. **(G)** Transmission electron microscopy showing the WT pollen. **(H)** Transmission electron micrograph showing the *Osfignl1* pollen. **(I)** A higher magnification image of the WT pollen wall from **(G)**. **(J)** A higher magnification image of the *Osfignl1* mutant pollen grain from **(H)**. Transmission electron micrographs of the WT **(K)** and *Osfignl1* mutant anther **(L)** at bicellular pollen stage. c, columella; f, foot layer; te, tectum; st, starch granules; T, tapetum; ML, middle layer; Bars = 20 μm **(A,D)**, 1 μm in **(C,F)**, 5 μm in **(B,E,G,H)**, 500 nm in **(I,J)**, and 5 μm in **(K,L)**.

The anther and microspore abnormalities between the wild-type and *Osfignl1* mutant were further analyzed by SEM and TEM observation. Mature pollen grains of the wild-type were spherical, larger, and filled with abundant starch granules, whereas *Osfignl1* pollen grains were irregular, shrunken, and failed to produce internal contents and nuclei (**Figures 3A,D,G,H**). The wild-type anther epidermis was less compact compared to the *Osfignl1* mutant (**Figures 3B,E**). In addition, the pollen exine of the wild-type was covered with sporopollenin which appeared typically granular, while in contrast, the *Osfignl1* pollen exine had irregular or “messy” sporopollenin deposits (**Figures 3C,F**). The typical pollen wall is composed of exine and intine, with exine divided into the tectum and foot layer. In the wild-type, mature pollen grains developed a normal tectum and foot layer, which were linked by the columella (**Figure 3I**), whereas *Osfignl1* pollen showed a thicker tectum and foot layer (**Figure 3J**). The thicker tectum and foot layer had an abnormal morphology and degenerated more than wild-type (**Figure 3J**), suggesting that mutation in the *Osfignl1* mutant also affects the pollen wall formation. TEM micrographs also showed that the tapetum and middle layer were swollen, and thicker than those of the wild-type at bicellular pollen stage. These observations suggest that the abnormal degradation of the tapetum occurs in *Osfignl1* during late anther development, which is probably an important reason for the abnormal anther maturation and sterility of pollen grains (**Figures 3K,L**).

### Meiosis Is Disrupted in the *Osfignl1* Mutant

**FIGURE 4:**
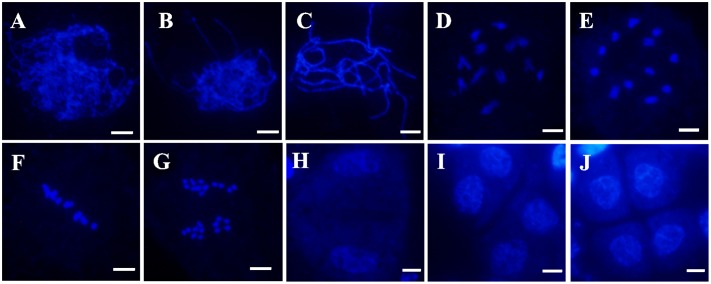
4′,6-diamidino-phenylindole (DAPI)-stained chromosome spreads of male meiosis in WT plants. **(A)** Leptotene. **(B)** Zygotene. **(C)** Pachytene. **(D)** Diplotene. **(E)** Diakinesis. **(F)** Metaphase I. **(G)** Anaphase I. **(H)** Dyad. **(I)** Telophase II. **(J)** Tetrad. Bars = 5 μm.

To determine whether pollen abortion resulted from defects in male meiosis, meiotic chromosomal behavior in male meiocytes of both wild-type and the *Osfignl1* mutant were examined by DAPI staining. In the wild-type, meiotic chromosomes began to condense and appeared as thin thread-like structures at leptotene (**Figure 4A**). At the zygotene stage, homologous chromosomes underwent synapsis and concentrated around the nucleolus on one side (**Figure 4B**). In wild-type plants, generation of thick, thread-like chromosomes were observed at pachytene, indicating that chromosomes were paired, and fully synapsed chromosomes were completed (**Figure 4C**). During diplotene, the synaptonemal complex disassembled and chiasmata, which linked the paired homologous chromosomes together, were visible (**Figure 4D**). In the wild-type, paired chromosomes condensed into 12 bivalents at diakinesis (**Figure 4E**). During metaphase I, the 12 highly condensed bivalents were aligned on the equatorial plate (**Figure 4F**). At anaphase I, homologous chromosomes separated and migrated in opposite directions of the cell (**Figure 4G**), finally leading to the formation of dyads at the end of meiosis I (**Figure 4H**). During the second meiotic division, the sister chromatids of each chromosome separated, similar to that in mitosis. After meiosis II, the tetrads of microspores were formed (**Figures 4I,J**).

**FIGURE 5:**
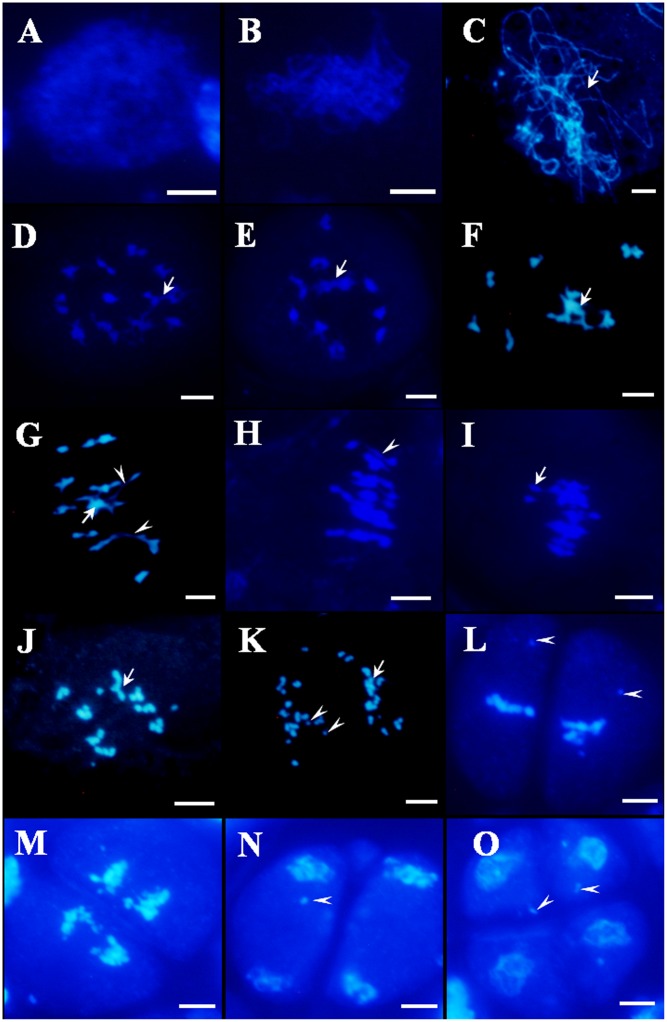
4′,6-diamidino-phenylindole-stained chromosome spreads of male meiosis in *Osfignl1* plants. **(A)** Leptotene. **(B)** Zygotene. **(C)** Pachytene. **(D,E)** Diplotene. Arrows indicate entangled chromosomes. **(F,G)** Diakinesis. Arrows indicate multivalent chromosome and the arrowheads indicate chromosome bridges. **(H,I)** Metaphase I, showing chromosome bridges (arrowhead). Chromosomes did not align on the equatorial plate (arrow). **(J,K)** Anaphase I, showing chromosome fragments (arrowheads) and entangled chromosome (arrows). **(L)** Metaphase II. **(M)** Anaphase II. **(N)** Telophase II. **(O)** Tetrad. From Metaphase II **(L)** to tetrad **(O)**, showing lagging chromosome (arrowheads). Bars = 5 μm.

In *Osfignl1* male meiocytes, meiotic chromosome behavior showed no obvious differences, compared with that of the wild-type from leptotene to zygotene (**Figures 4A,B**, **5A,B**). However, chromosomal abnormalities appeared from the pachytene to the tetrad stage. Chromosomes appearing as single threads were observed at the pachytene stage (**Figure 5C**). At diplotene, abnormal chromosomes were linked together, and we observed abnormally shaped chromosomes, instead of well-defined bivalents (**Figures 5D,E**). During diakinesis, irregularly shaped chromosomes, chromosome bridges, and entangled chromosomes were visualized (**Figures 5F,G**). In metaphase I, most of the entangled chromosomes were aligned on the equatorial plate, such as those found in the wild-type, while other chromosomes did not align at all on the equatorial plate (**Figure 5I**). Chromosome bridges also were found in *Osfignl1* at metaphase I (**Figure 5H**). At anaphase, the chromosomes separated and moved to opposite sides of the cell, because of these chromosome fragments, the numbers of chromosomes were difficult to distinguish as separate chromosomes (**Figure 5J**). Moreover, multiple chromosomes and several chromosome fragments were observed at this stage (**Figure 5K**). During the second meiotic division, lagging chromosomes and chromosome fragments were still observed in the cell, and resulted in formation of the tetrads with nuclei of different sizes from wild-type (**Figures 5L–O**). These results indicated that unrepaired ectopic recombination may occur between non-homologous chromosomes, finally leading to chromosome breakage and ultimately abortion of microspore development. These results suggest that meiotic defects cause complete male sterility of *Osfignl1* plants.

We compared meiosis progression between the wild-type and *Osfignl1* male meiocytes based on the lengths of spikelets. In *Osfignl1* mutant plants, male meiocytes progressed normally before the pachytene phase as wild-type did. In the wild-type, 52% of microsporocytes were found in 5.6–5.8 mm spikelets during the diplotene stage, while 90% of *Osfignl1* mutant microsporocytes were still in the pachytene stage (Supplementary Figure S3). When most of wild-type meiocytes (94%, *n* = 62) in relatively longer (6.3–6.5 mm) spikelets had progressed from pachytene into late prophase I, the *Osfignl1* mutant meiocytes (100%, *n* = 187) remained in the pachytene phase (Supplementary Figure S3). When spikelet length reached 6.7–6.8 mm, a majority of wild-type meiocytes completed meiosis, however, 5% of *Osfignl1* male meiocytes were still observed at the pachytene stage. These observations suggest that a slowdown of the pachytene stage of meiosis in *Osfignl1* meiocytes leads to a lengthening of the progression of male meiosis.

### Map-Based Cloning of *OsFIGNL1* and Its Protein Sequence Analysis

**FIGURE 6:**
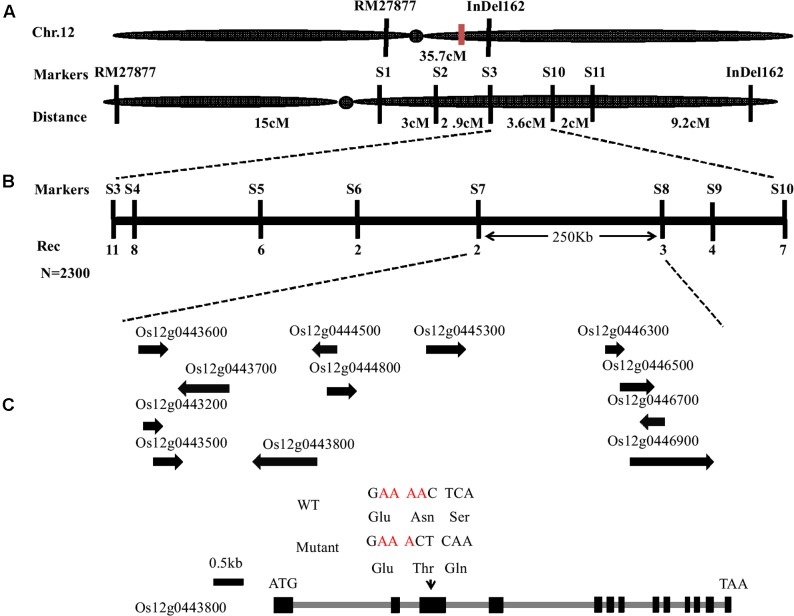
Map-based cloning of *OsFIGNL1* gene on chromosome 12. **(A)** Primary mapping was performed using markers RM27877 and InDel162. **(B)** The *OsFIGNL1* locus was finally mapped to a 250-kb region between the molecular markers S7 and S8. Names and positions of the markers are noted. **(C)** A schematic representation of the gene structure of *OsFIGNL1*. The mutant sequence has a single nucleotide deletion in the third exon. ATG and TAA represent the start and stop codon, respectively. Black boxes indicate exons, and intervening gray lines indicate introns. cM, Centimorgan; Rec, Recombinants; N, Total number of F_2_ plants used for fine mapping.

To isolate the *OsFIGNL1* gene, a map-based cloning strategy was employed. We generated an F_2_ population by crossing the *Osfignl1* mutant with Zhonghua11 (*japonica* rice) for linkage analysis. The mutant gene was primarily mapped to a genetic distance of 22.9 and 12.8 cM with RM27877 and InDel162 markers on chromosome 12, respectively (**Figure 6A**). To finely map the *OsFIGNL1* gene, a total of 2,300 F_2_ population individuals exhibiting the male-sterile phenotype were used for genetic analysis. Finally, we mapped the candidate gene to a 250 kb between markers S7 and S8, which contained 12 predicted open reading frames, annotated by the Rice Annotation Project Database (RAP-DB^5^) (**Figure 6B**). We sequenced the predicted genes of this 250-kb region, and found that the *Osfignl1* mutant carries a single nucleotide Adenine deletion in the third exon of the annotated gene *Os12g0443800.1*, resulting in a premature stop codon at the 187th amino acid residue (**Figure 6C**). Based on the annotations of the RAP-DB, we found that *Os12g0443800.2* and *Os12g0443800.1* were annotated as a P-loop containing nucleoside triphosphate hydrolase superfamily protein with putative, overlapping transcribed sequences (Supplementary Figure S4A), suggesting that these ORFs may be a single, transcriptional unit. As expected, RT-PCR showed that *Os12g0443800* containing *Os12g0443800.1* and *Os12g0443800.2* represented a single mRNA, and the sequence of *Os12g0443800.2* revealed a mis-annotation in the RAP-DB. *OsFIGNL1* consisted of 13 exons and 12 introns (**Figure 6C**), comprising a 2,085-bp cDNA region, encoding a predicted protein of 694 amino acids (Supplementary Figure S4B).

To confirm that the male sterility was caused by the mutation in Os12g0443800, we amplified an 11.8-kb genomic DNA fragment containing the full-length ORF of *Os12g0443800* from Zh8015, and transformed it into calli induced from young panicles of homozygous *Osfignl1* mutant plants. The transgenic plants had high seed-setting rate, similar to those of wild-type (Supplementary Figure S5A). The complemented lines accumulated abundant starch granules and displayed yellow anthers (Supplementary Figures S5B–E), indicating that the 1 bp deletion in *Os12g0443800* was responsible for the pollen abortion phenotype of *Osfignl1*. To further confirm *OsFIGNL1* function is responsible for the phenotype observed in the *Osfignl1* mutant, we generated CRISPR/Cas9 mutation lines of *OsFIGNL1* in the Zh8015 genetic background. Among the 12 CRISPR T_0_ lines obtained, two lines were found to harbor a homozygous mutation in *OsFIGNL1* with significantly reduced transcription level (Supplementary Figures S6A,E). As expected, the two homozygous lines phenotypically mimicked *Osfignl1* with shorter, thinner anthers and shriveled pollens, while the remaining heterozygous or negative lines grew normally as wild-type (Supplementary Figures S6B,C). Similar to the *Osfignl1* mutants, aberrant chromosomes and chromosomal entanglement also can be found in the homozygous mutant lines during male meiosis (Supplementary Figure S6D). Taken together, the results above demonstrated that *Os12g0443800* was the rice *OsFIGNL1* gene.

An interProScan search found that *OsFIGNL1* was predicted to encode a protein with an ATPase domain (453–583) and a VPS4 domain (656–690)^6^ (Supplementary Figure S4B). Further alignment of the protein sequences of FIGNL1 homologs from *Arabidopsis thaliana* (Genbank accession KM055500), *O. sativa* (Os12g0443800), *Homo sapiens* (NP_001036227.1), *Mus musculus* (NP_001156832.1), and *C. elegans* (NP_504197.1) indicated that all five proteins contained a VPS4 domain and an ATPase domain (Supplementary Figure S7). We constructed a phylogenetic tree from the OsFIGNL1 full-length protein sequences of 23 orthologs from different species acquired by BLASTP (NCBI) search using rice OsFIGNL1 as the query. OsFIGNL1-related sequences were classified into two clades: clade I is comprised of dicot proteins, while the clade II members belong to monocotyledon species including the *Brachypodium distachyon*, *Hordeum vulgare*, *Zea mays*, *Setaria italica*, and *Sorghum bicolor* (Supplementary Figure S8). The BLASTP search also revealed that OsFIGNL1 was closely related to *Brachypodium distachyon* with 80% identity. Taken together, these analyses imply that OsFIGNL1 is conserved in land plants.

### Subcellular Localization *OsFIGNL1* Protein and Expression Pattern of Rice *OsFIGNL1* Gene

**FIGURE 7:**
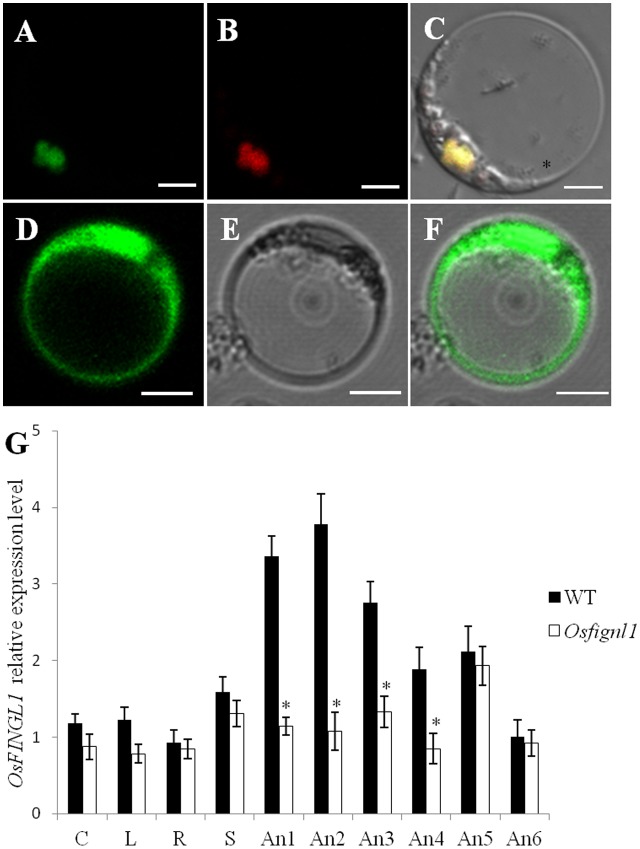
Subcellular localization and expression pattern analysis of rice *OsFIGNL1*. **(A)** Subcellular localization of full-length OsFIGNL1 fused with GFP is detected in nucleus. **(B)** OsMADS3-mCherry was used as a nuclear marker. **(C)** A merged image of **(A,B)**. **(D–F)** GFP alone is detected throughout the rice protoplast cells. Bars = 5 μm. **(G)** Expression pattern analysis of rice *OsFIGNL1*. R, mature root; C, mature culm; L, mature leaves; S, mature sheath. An1, spikelet lengths from 4 to 5 mm; An2, spikelet lengths from 5 to 6 mm; An-3, spikelet lengths from 6 to 7 mm; An-4, spikelet lengths from 7 to 8 mm; An-5, spikelet lengths from 8 to 9 mm; An-6, spikelet lengths > 10 mm. *OsActin1* was used a control for normalizing expression in all assays. ^∗^Indicate significant difference at *P* < 0.01.

In order to determine the subcellular localization of OsFIGNL1 protein, we constructed a vector expressing OsFIGNL1-GFP (green fluorescent protein) fusion protein driven by the *35S* promoter. After introduction into rice protoplast cells, the OsFIGNL1-GFP fusion protein was detected exclusively in the nucleus (**Figures 7A–C**), whereas the GFP empty vector control was found throughout the cell (**Figures 7D–F**), indicating that *OsFIGNL1* encodes a nuclear-localized protein. qRT-PCR analysis was conducted to examine the expression patterns of *OsFIGNL1* in various tissues and growth stages. The results suggested that *OsFIGNL1* was constitutively expressed in all the tested issues, including mature root, mature sheath, mature leaf, mature culm, relatively higher levels detected in anthers. Particularly, *OsFIGNL1* expressed predominantly in anthers at the meiosis stage, and progressively reduced as anther development proceeded (**Figure 7G**). This expression pattern well supported the compromised meiosis that we observed in *Osfignl1*.

### OsFIGNL1 Interacts with Rice RAD51 and DMC1

**FIGURE 8:**
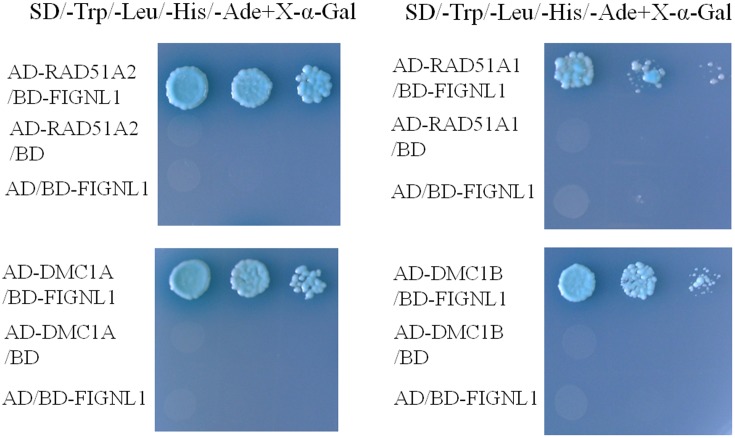
Yeast two-hybrid interactions between rice OsFIGNL1 and RAD51A1 RAD51A2, DMC1A and DMC1B. Y2H assay to test interactions between OsFIGNL1, RAD51A1, RAD51A2, DMC1A and DMC1B. *OsFIGNL1* was cloned into the bait vector pGBKT7 (BD), and rice *RAD51A1*, *RAD51A2*, *DMC1A*, and *DMC1B* were inserted in the pray vector pGADKT7 (AD). The empty pGBKT7 and pGADKT7 vectors were used as negative controls.

**FIGURE 9:**
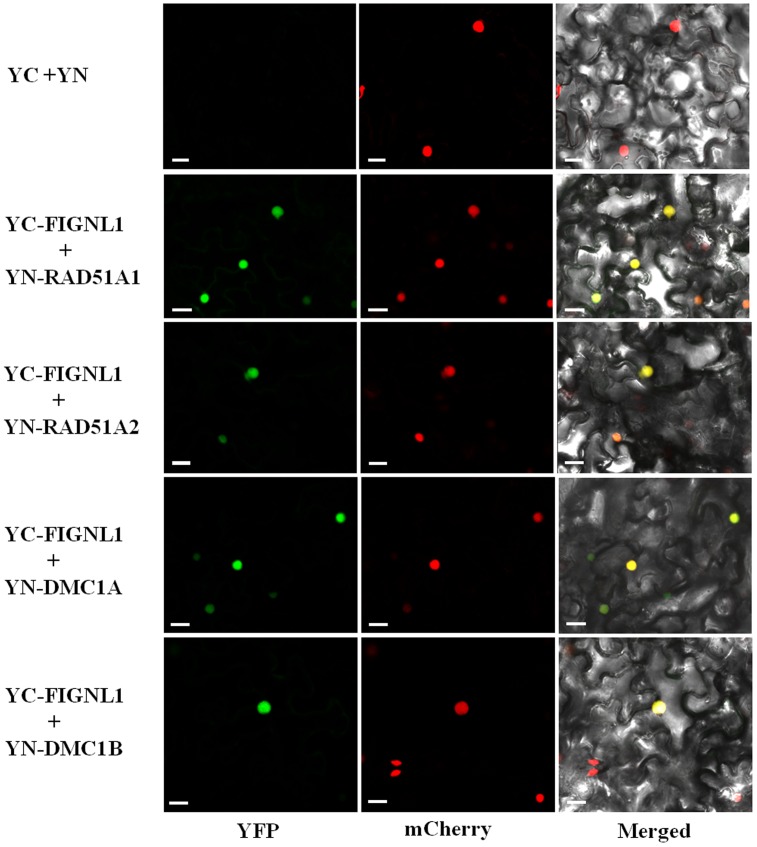
Bimolecular fluorescence complementation (BiFC) assays of rice OsFIGNL1 interaction between RAD51A1, RAD51A2, DMC1A, and DMC1B *in vivo*. The expression of pSPYNE (YN)/pSPYCE(YC) was used as negative control. OsMADS3-mCherry was used as a nuclear marker. Bars = 20 μm.

A previous study showed that human FIGNL1 specifically interacts with RAD51 protein (Yuan and Chen, 2013). Rice genome contains two highly similar RAD51 proteins, *RAD51A1* and *RAD51A2*, both of which are DNA-binding and participate in homologous chromosome pairing, though the specific activity may differ from each other (Morozumi et al., 2013). To investigate whether OsFIGNL1 interacts with RAD51A1, RAD51A2, DMC1A, and DMC1B in rice, we cloned full-length *RAD51A1*, *RAD51A2*, *DMC1A*, and *DMC1B* into the prey vector pGADT7, while *OsFIGNL1* was cloned into the bait vector pGBKT7. Yeast two-hybrid analysis demonstrated that rice OsFIGNL1 could physically interact with the rice RAD51A1, RAD51A2, DMC1A, and DMC1B, but not with RAD51B and RAD51D (data not shown) (**Figure 8**). To prove the interactions further, a BiFC assay demonstrated that OsFIGNL1 interacted with RAD51A1, RAD51A2, DMC1A, and DMC1B in *Nicotiana benthamiana* leaves cells and interacting proteins had a nuclear localization. These results suggest that rice OsFIGNL1 interacts with rice RAD51A1, RAD51A2, DMC1A, and DMC1B *in vivo* (**Figure 9**).

## Discussion

### OsFIGNL1 Is a Member of AAA Family and Controls Male Fertility in Rice

In the present study, using map-based cloning, we isolated the rice fertility gene *OsFIGNL1*. The results of this mutagenesis and complementation study strongly indicated that *OsFIGNL1* is a critical gene for male fertility in rice. The morphological and cytologic analyses suggest that OsFIGNL1 plays a key role in anther development, and is essential for male meiosis. Here, we report that the rice *OsFIGNL1* gene encodes a nuclear protein which belongs to a group of meiosis-related AAA proteins (Frickey and Lupas, 2004). FIDGETIN is the first reported AAA protein, which contains a conserved ATPase domain spanning 200–250 amino acid residues. Sequence alignment showed that the FIGNL1 protein consists of several motifs including a VPS4 domain (Vajjhala et al., 2006), FIGNL1-RAD51-Binding Domain (FRBD) (Yuan and Chen, 2013), as well as the conserved Walker A, Walker B, and Arg fingers domains of the ATPase domain (Yakushiji et al., 2004; Peng et al., 2013), indicating that these functional domains were evolutionarily conserved and likely critical for its function in these species. However, despite of the high level of conservation in the ATPase region, the N-termini were not conserved among the different species of OsFIGNL1 homologs (Supplementary Figure S7). Furthermore, human FIGNL1’s RAD51 Binding Domain (FRBD), a region that is highly specific for binding RAD51, is also found in *O. sativa* (Supplementary Figure S7) and *Arabidopsis* (Girard et al., 2015). Several FIGNL1 proteins have been reported to be functional in regulating diverse cellular processes among different species. Although there may exist essential and conserved function domains among FIGNL1s, molecular and biological functions of the AAA family members may have diverged among different species. For example, Zhao et al. (2016) recently found that human FIGNL1 localizes at centrosomes, and is involved in ciliogenesis. Previous reports showed that *C. elegans* FIGNL1 binds to microtubules and controls mitotic progression in the germ line (Luke-Glaser et al., 2007). In our study, the results revealed that the loss function of OsFIGNL1 does not affect female fertility, but leads to complete male sterility. This conclusion was supported by backcrossing between the *Osfignl1* mutant and wild-type plants using a mutant as maternal recipient, which also can produce hybrid seeds. The phenotypic characterization of embryo sac development of differential stages showed that *Osfignl1* embryo sacs had no obvious differences compared with wild-type plants, suggesting that OsFIGNL1 is only essential for male meiosis but not indispensable for female reproductive development. In contrast to most of the previously reported meiotic mutants which exhibited complete male and female sterility, the *Osfignl1* mutant is male sterile with normal female fertility. These findings suggest that the meiosis in rice megagametogenesis and male gametogenesis are regulated by different genes. Since *OsFIGNL1* is most highly expressed in meiosis stage anthers, though its expression is not limited to the anther, we hypothesize that OsFIGNL1 plays a minor role in female meiosis aside from its important role in male meiosis. This role may be associated with regulation of gene expression in different tissues. Here, the present results demonstrate that the rice OsFIGNL1 mainly functions in male fertility and is essential for male meiosis.

### OsFIGNL1 May Be Involved in the Interplay between the Rice RAD51 and DMC1 for Repair Meiotic DSBs

RAD51 and DMC1, both highly conserved in eukaryotes, load onto single-stranded DNA and catalyze the strand exchange between homologous DNA molecules (Baumann and West, 1998). RAD51 protein was shown to mediate the search for homologous sequences and catalyze strand exchange, which plays an import role in DSBs repair (Baumann et al., 1996; Sheridan et al., 2008; Kurzbauer et al., 2012). *S. cerevisiae* RAD51, similar to *Escherichia coli* RecA protein, plays an essential role in mitotic and meiotic homologous recombination (Shinohara et al., 1992; Krogh and Symington, 2004). In *Arabidopsis*, the loss of function of AtRAD51 causes severe abnormalities in meiosis and complete sterility, although the mutant exhibits normal vegetative and flower development (Li et al., 2004). *Arabidopsis Atrad51* null mutant is deficient in DSBs repair and exhibits extensive chromosome fragmentation and chromosome bridges during meiosis, whereas *Atdmc1* single mutant shows 10 univalents at metaphase I (Pradillo et al., 2012). The phenotypic differences between *Atdmc1* and *Atrad51* mutants suggest that the specific role of AtRAD51 and AtDMC1 in plant meiosis may be different. Previous studies suggest that AtRAD51 seem to be more involved in intersister recombination, while AtDMC1 could be primarily repair meiotic DSBs using the homologous chromosomes as templates (Pradillo et al., 2012). Alternatively, Kurzbauer et al. (2012) proposed that AtRAD51 and AtDMC1 localize to opposite DNA ends at a meiotic DSB, and AtDMC1 functions independently and spatially separated from AtRAD51 during meiosis. Recently, Da Ines et al. (2013) have reported that AtDMC1 would be chiefly responsible for catalyzing strand invasion, while AtRAD51 would just play a supporting role for promoting AtDMC1 activity in meiotic homologous recombination. In rice, *Osdmc1a Osdmc1b* mutant displays univalent chromosomes at diakinesis and synapsis is seriously disrupted (Wang H.J. et al., 2016), resulting in complete sterility. However, the meiotic phenotype has not been reported in rice *rad51a1*, *rad51a2*, and *rad51a1 rad51a2* double mutant to date. A BLASTP (NCBI) search revealed that the rice OsFIGNL1 was similar to the *Arabidopsis* FIGNL1 with 60% identity (Supplementary Figure S7). So far, only *Arabidopsis FIGNL1* has been well characterized in plants (Girard et al., 2015). In wild-type *Arabidopsis* meiosis, FIGNL1 limits the formation of COs by regulating AtRAD51 and AtDMC1 foci dynamics, while mutation of *FIGNL1* increases the number of AtRAD51 foci, but not AtDMC1 foci. In *Arabidopsis*, mutation of *FIGNL1* would restore the level of CO formation in CO-deficient mutants. In addition, human FIGNL1 is found to interact with RAD51, and is necessary for homologous recombination repair (Yuan and Chen, 2013). In this study, using a yeast two-hybrid system and BiFC assay, we revealed strong interactions of OsFIGNL1 with rice RAD51A1, RAD51A2, DMC1A, and DMC1B. Given their crucial roles in strand exchange activities during meiosis, OsFIGNL1 is likely to be involved in homologous recombination mediated by the rice RAD51 and DMC1 proteins. *In vivo* data have shown that AtBRCA2 protein could bind to AtDMC1 and AtRAD51 (Dray et al., 2006). AtBRCA2 mediates the recruitment of AtRAD51 and AtDMC1 for strand invasion and plays an important role for both somatic and meiotic homologous recombination (Seeliger et al., 2012). It is proposed that OsFIGNL1 may work as a complex with DMC1 and RAD51 for recruitment to DSBs at the initiation of meiotic recombination and thus participates in meiotic homologous recombination in rice. It seems likely that OsFIGNL1 may act as an accessory factor involved in DNA strand invasion by cooperating with DMC1/RAD51 regulating male meiosis. However, cytological studies and biochemical data are needed to establish the molecular mechanism of the effect of OsFIGNL1 on DMC1/RAD51, which load onto chromosomes for DNA strand invasion in accurate meiotic recombination.

### The Loss of OsFIGNL1 Function Disturbs Meiotic Chromosome Behavior and Affects Meiotic Cell Cycle Progression

In *Arabidopsis*, several meiotic mutants, such as *Atrad51*, *Atrad51c*, and *Atxrcc3*, have been found to show observable chromosome fragments or chromosome entanglement during meiosis (Pradillo et al., 2014). Previous studies found that *Arabidopsis Atrad51* mutants failed to synapse and instead developed an entangled mass of chromosomes, interconnected by chromatin bridges at metaphase I (Li et al., 2004; Pradillo et al., 2012). The abnormal DSB repair mutants that exhibit various meiotic defects of chromosome fragmentation also have been reported in rice. The *Osmre11* mutant contained an entangled mass of chromosomes at diakinesis, and formed large chromosome aggregates at metaphase I (Ji et al., 2013). The rice XRCC3 and OsRAD51C have been reported to be essential for DSB repair in meiosis (Tang et al., 2014; Zhang et al., 2015), and their mutants exhibit chromosome fragments and chromosome entanglements during the process of meiosis. In the *Osrad51c* mutant, chromosome fragments were first observed at early metaphase I (Kou et al., 2012; Tang et al., 2014). In the rice *xrcc3-1* meiocytes, chromosomes with irregular shape and chromosome entanglements were observed from diplotene to metaphase I (Zhang et al., 2015). A previous study has shown that chromosomal fragmentation is thought to be caused by the accumulation of unrepaired DSBs of DNA (Schommer et al., 2003). In the *Osfignl1* mutant, the entangled chromosomes and chromosome bridges were observed at diakinesis and metaphase I, possibly due to the defects in homologous recombination or improper repair of DSBs. Compared with the previously reported *Osrad51c* mutants and the *xrcc3* mutant (Tang et al., 2014; Zhang et al., 2015), the abnormal chromosome behavior phenotype in the *Osfignl1* mutant, in our study, was less severe from metaphase I to the end of the meiotic stage. However, it is obvious that the chromosome morphology among these mutants was not the same. PMCs of *Osfignl1* mutant displayed obvious defects in meiotic chromosome behavior, which resulted in complete male sterility due to pollen abortion. The meiotic defects were visible in *Osfignl1* male meiocytes starting from pachytene stage. We also noted that some chromosomes were trapped as single threads at the pachytene stage, suggesting that homolog juxtaposition and synapsis may be deficient. In the diakinesis stage, chromosome bridges between two chromosomes were observed in the PMCs of *Osfignl1* plants, which might be associated with unresolved homologous/non-homologous intermediates during the first meiotic division. We also observed chromosome fragmentation and lagging chromosomes in *Osfignl1* male gametocytes from late prophase I to meiosis II, which suggests that mutation of OsFIGNL1 causes broken chromosomes or incorrect segregation of homologous chromosomes.

Loss of OsFIGNL1 function in mice leads to an impaired progression of the germ cells through lengthening the first wave of pachytene stages, thus affecting meiotic cell cycle progression (L’Hote et al., 2011). The extension of the pachytene during the meiosis I in the *Osfignl1* male meiocytes is consistent with the role of FIGNL1 in the regulation of male meiosis in mice (L’Hote et al., 2011). However, in *Arabidopsis*, different mutation sites of the *FIGNL1* gene allow formation of bivalents without affecting meiotic cell cycle progression (Girard et al., 2015), which is inconsistent with the observation that *Osfignl1* stayed longer at the pachytene stage than those in wild-type meiocytes. A reason for abnormal progression of the meiotic cell cycle in *Osfignl1* male meiocytes may be that our mutagenesis of the rice *OsFIGNL1* gene created a premature stop at the 187th amino acid residue, leading to a severe alteration of the protein structure, which differs from previously studied mutations of *Arabidopsis FIGNL1*. In *Arabidopsis*, loss of function of the synaptonemal complex protein ZYP1 significantly delays prophase I progression, indicating the existence of surveillance mechanisms that monitor progression through prophase I (Higgins et al., 2005). It is possible that prolonged arrest in the pachytene stage is the result of the existence of a recombination checkpoint in *Osfignl1* mutant. The pachytene checkpoint was found to be activated in mouse (Wang et al., 2011), *C. elegans* (Gartner et al., 2000) and *Drosophila* (Ghabrial and Schupbach, 1999). However, meiotic mutants that invoke the pachytene checkpoint control mechanisms were less studied in plants. The observation that male meiocytes in *Osfignl1* can proceed through late prophase I to the end of meiosis II after a brief arrest. Taken together, these results suggest that OsFIGNL1 participates in the regulation of meiotic cell cycle progression and plays an important role in recombination events during male meiosis in rice.

## Conclusion

This report presents a functional characterization of *OsFIGNL1* in rice. Our results show that *OsFIGNL1* encodes a conserved AAA-ATPase domain and participates in control of microspore and anther development in rice. Our results provide evidence that disruption of OsFIGNL1 function leads to extension of meiotic cell cycle progression and abnormalities in chromosome behavior during male meiosis, thus resulting in pollen abortion. This work provides novel insight into the function of OsFIGNL1 in rice meiosis.

## Author Contributions

Study conception and design: PZ, YZ, LC, and SC; acquisition of data: PZ, LS, SS, and WW; analysis and interpretation of data: PZ, YZ, ZL, DX, and PY; drafting of manuscript: PZ, YZ, ZY, BS, KW, and LC; critical revision: PZ, YZ, SC, and LC. All authors have read and approved to submit it to your journal.

## Conflict of Interest Statement

The authors declare that the research was conducted in the absence of any commercial or financial relationships that could be construed as a potential conflict of interest.

## Footnotes

1 http://www.cambia.org

2 http://www.ncbi.nlm.nih.gov

3 http://www.genome.jp/tools/clustalw/

4 http://www.cambia.org

5 http://rapdb.dna.affrc.go.jp/

6 www.ebi.ac.uk/interpro/search/sequence-search
