# Supplementary material for: The Rice AAA-ATPase OsFIGNL1 Is Essential for Male Meiosis
Source: Front Plant Sci. 2017 Sep 27;8:1639. doi: 10.3389/fpls.2017.01639 (PMC5624289; doi:10.3389/fpls.2017.01639)
Supplement: Supplementary file 1 [file Presentation_1.PDF]

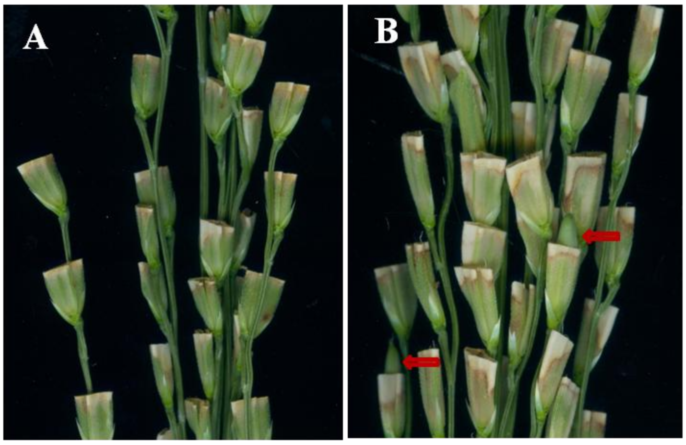

Supplementary Figure S1. Female fertility analysis in the *Osfignl1* mutant. (A) The *Osfignl1* mutant without pollination. (B) The *Osfignl1* mutant is pollinated with wild-type pollen grains.

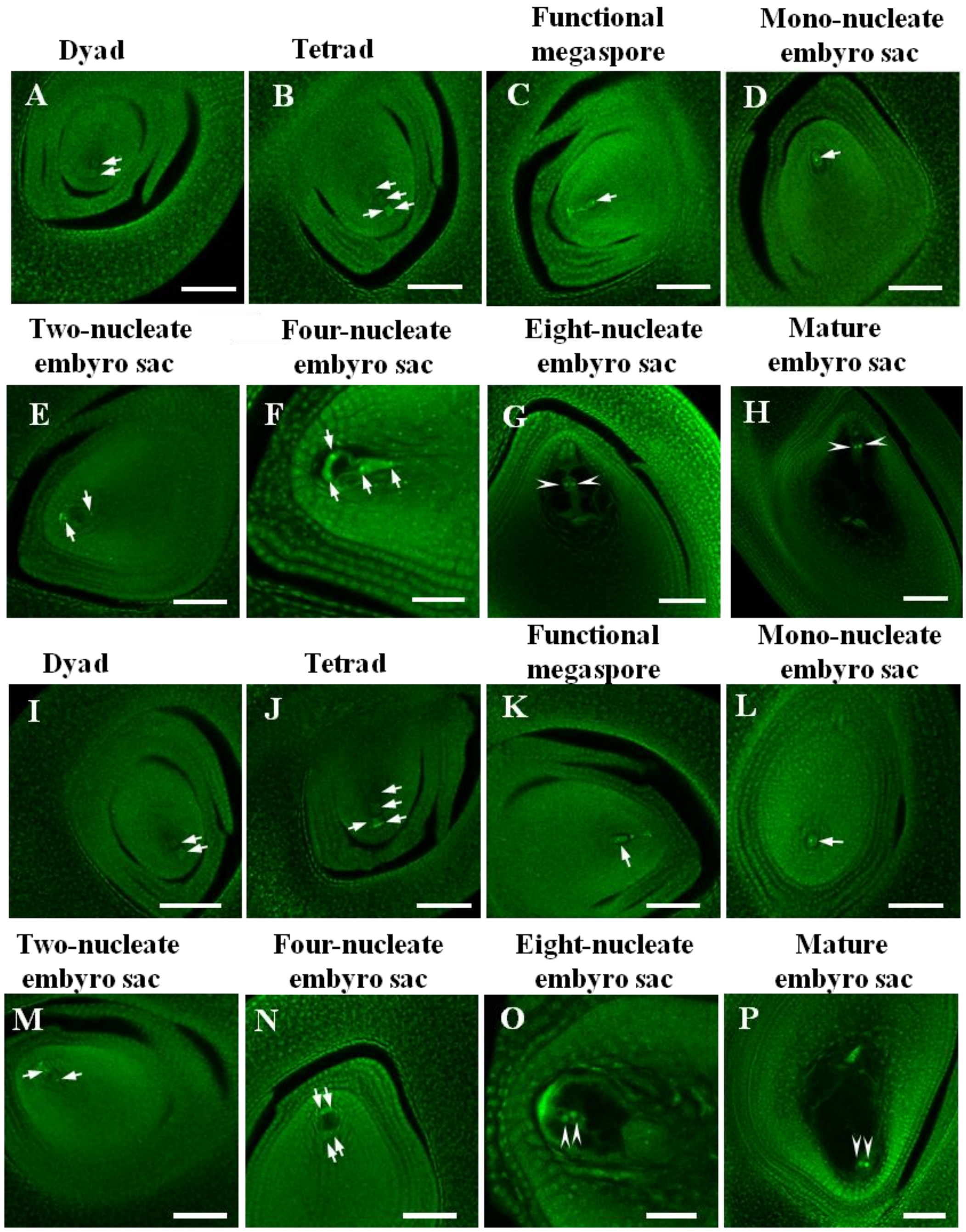

Supplementary Figure S2. Development of the embryo sac in the wild type (A–H) and *Osfignl1* mutant (I–P). Nuclei during megasporogensis (arrows), and megagametogenesis (arrowhead). Bars = 50 μm.

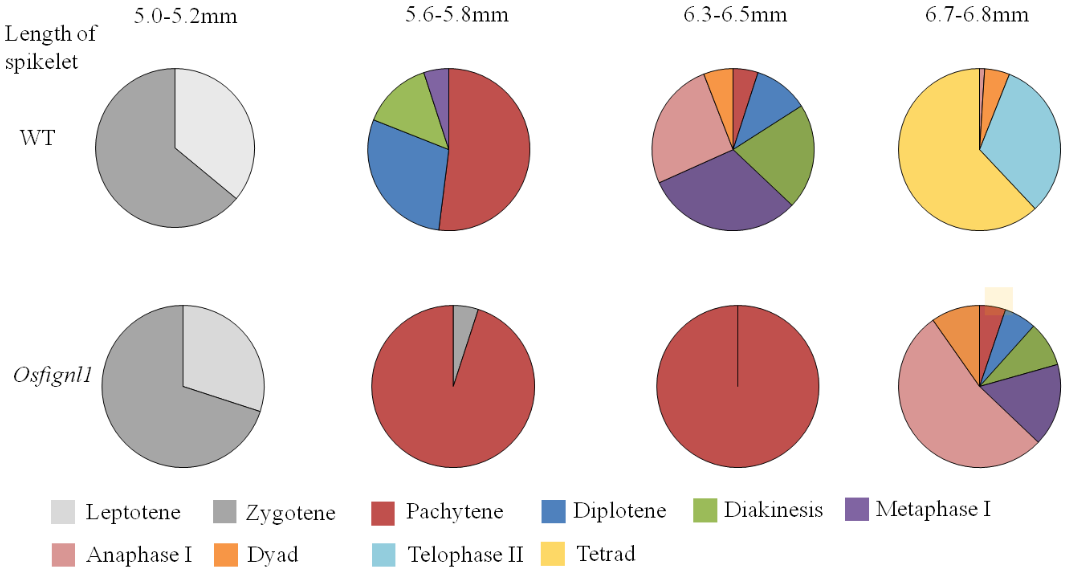

Supplementary Figure S3. Statistical results of meiotic stages in both the wild type (WT) and *Osfignl1* developing spikelets. In wild type, pachytene cells mainly (52%) exist in 5.6-5.8 mm spikelets. In *Osfignl1*, spikelets length at 5.8-6.5 mm showing the pachytene stage (90%-100%). The meiotic cell cycle is probably delayed at the pachytene-diplotene transition.

A

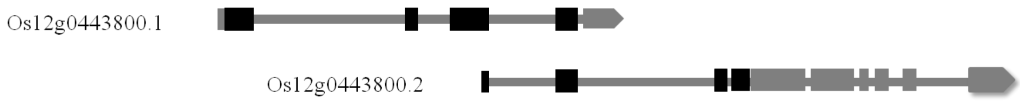

B

MAEQSHAGDGGGGGAGSGEPTNWRKEADDRLRRLHSLQFGADVALEGKDPAGAQVLALRL 60

LGFLDSQALPGDGGAAGHEASFVAPIRAAASSSVAAAIRARAGRSDSAVFKLAEKDVGCV 120

FAKTGEVNIEKIKCSKYFQALLQKSKGHAAEQMKTTDCQESTIEEGPHVEENSTDMENEK 180

LSIRASKLVMQRKLTSLRSHKPLKANVVQDGNMFKSVSNISNESVAVENGVRTNHTDNKY 240

TAYMDLEDDDRPRGLLQNAKRKHAGFRSPICEHANSPLSNDEADAPANEFVTARTKLEMD 300

SVQKYGHNGTQGASVSPQCDNNPNNRNYGVRPTWNSRRGLRGNFVPPIRNNGGSTSNMTS 360

RVIGKNDDSMGDSTRKCLEMLCGPDGELPEKLRNLEPRLIEHVSNEIMDKDPNVRWDDIA 420

GLEHAKKCVTEMVIWPLLRPDIFRGCRSPGRGLLLFGPPGTGKTMIGKAIAGEAKATFFY 480

ISASSLTSKWIGEGEKLVRALFGVACCRQPAVIFVDEIDSLLSQRKSDGEHESSRRLKTQ 540

FLIEMEGFDSGNDQILLIGATNRPQELDEAARRRLTKRLYIPLPSSEARAWIIRNLLEKD 600

GLFKLTEEETNIVCKLTEGYSGSDMKNLVKDASMGPLREALQQGVGITKLNKEDMRPVML 660

KDFETALQEVRPSVSSSELGTYEEWNRQFGSLAN 694

Supplementary Figure S4. Amino acid sequence of OsFIGNL1 protein. (A) The gray boxes are the predicted 5′UTR and 3′UTR in the RAP-DB (http://rapdb.dna.affrc.go.jp/). (B) The amino acid sequence of OsFIGNL1. The core ATPase conserved domain is underlined. VPS4 domain is double underlined.

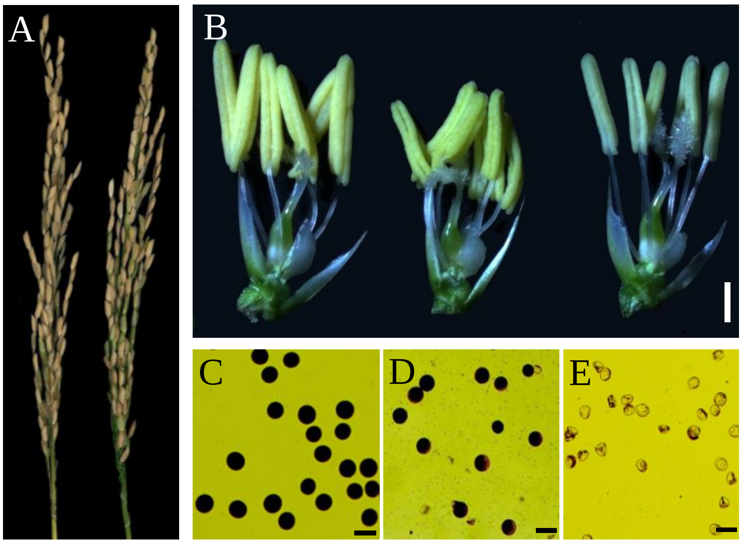

Supplementary Figure S5. Phenotype analysis of complementation of the *Osfignl1* mutant. (A) Phenotypes of the wild type panicle (left) and the complemented panicle (right) at the harvest stage. (B) Phenotypes of the flowers from a wild-type (left), the complemented line (middle) and a *Osfignl1* (right). (C) I_2–KI pollen grains staining of the wild type. (D) I_2–KI pollen grains staining of the complemented line. (E) I_2–KI pollen grains staining of the *Osfignl1* mutant. Bars = 1 mm in (B), and 50 µm in (C), (D) and (E).

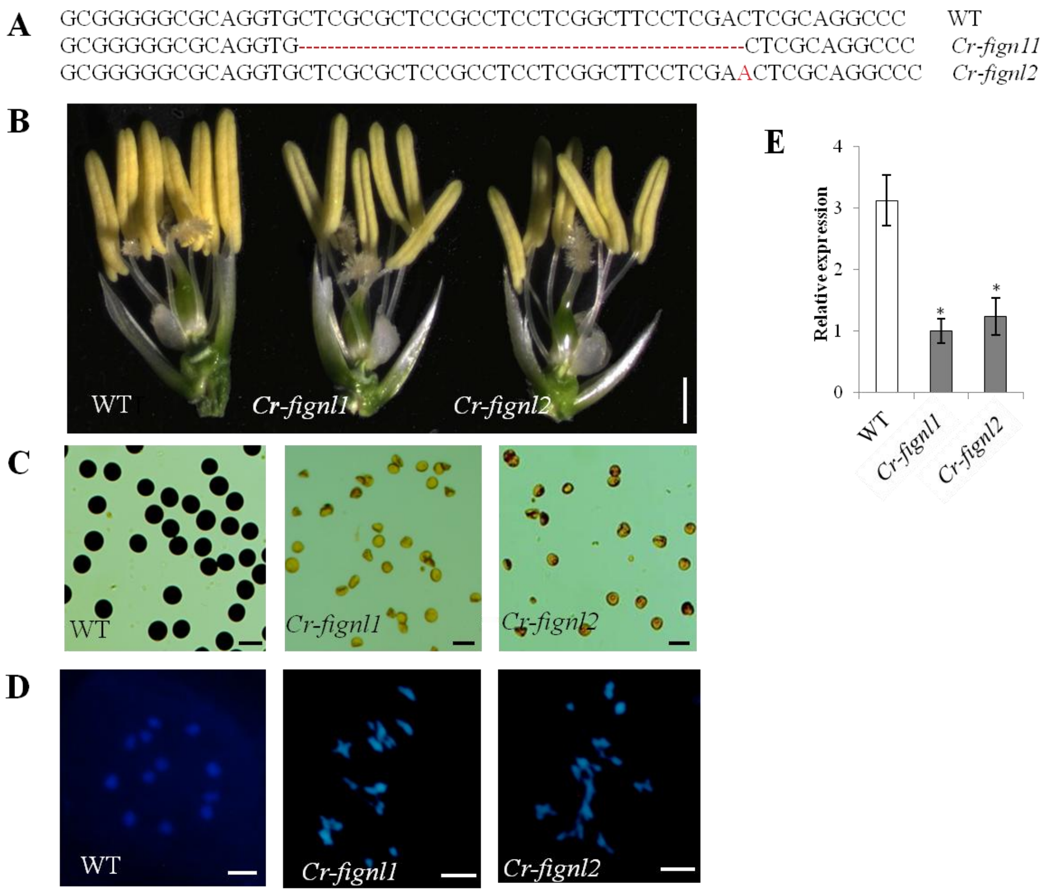

Supplementary Figure S6. CRISPR/Cas9-induced *Osfignl1* mutant plants and phenotype analysis. Target site of CRISPR/Cas9-mediated target mutagenesis in the rice *OsFIGNL1* gene. (A) The sgRNA target regions of different mutant lines are shown in red. (B) Comparison of wild-type (left), *Cr-fignl1* (middle) and *Cr-fignl2* (right) anther. (C) Comparison of wild-type (left), *Cr-fignl1* (middle) and *Cr-fignl2* (right) pollen. (D) Phenotypes of male meiocytes in a wild-type (left), *Cr-fignl1* (middle) and *Cr-fignl2* (right) at diakinesis. (E) qRT-PCR analysis of *OsFIGNL1* in wild-type, *Cr-fignl1* and *Cr-fignl2* at the meiosis stage (spikelet lengths from 5-6 mm). Bars = 1mm in (B), 50 µm in (C) and 5µm in (D).

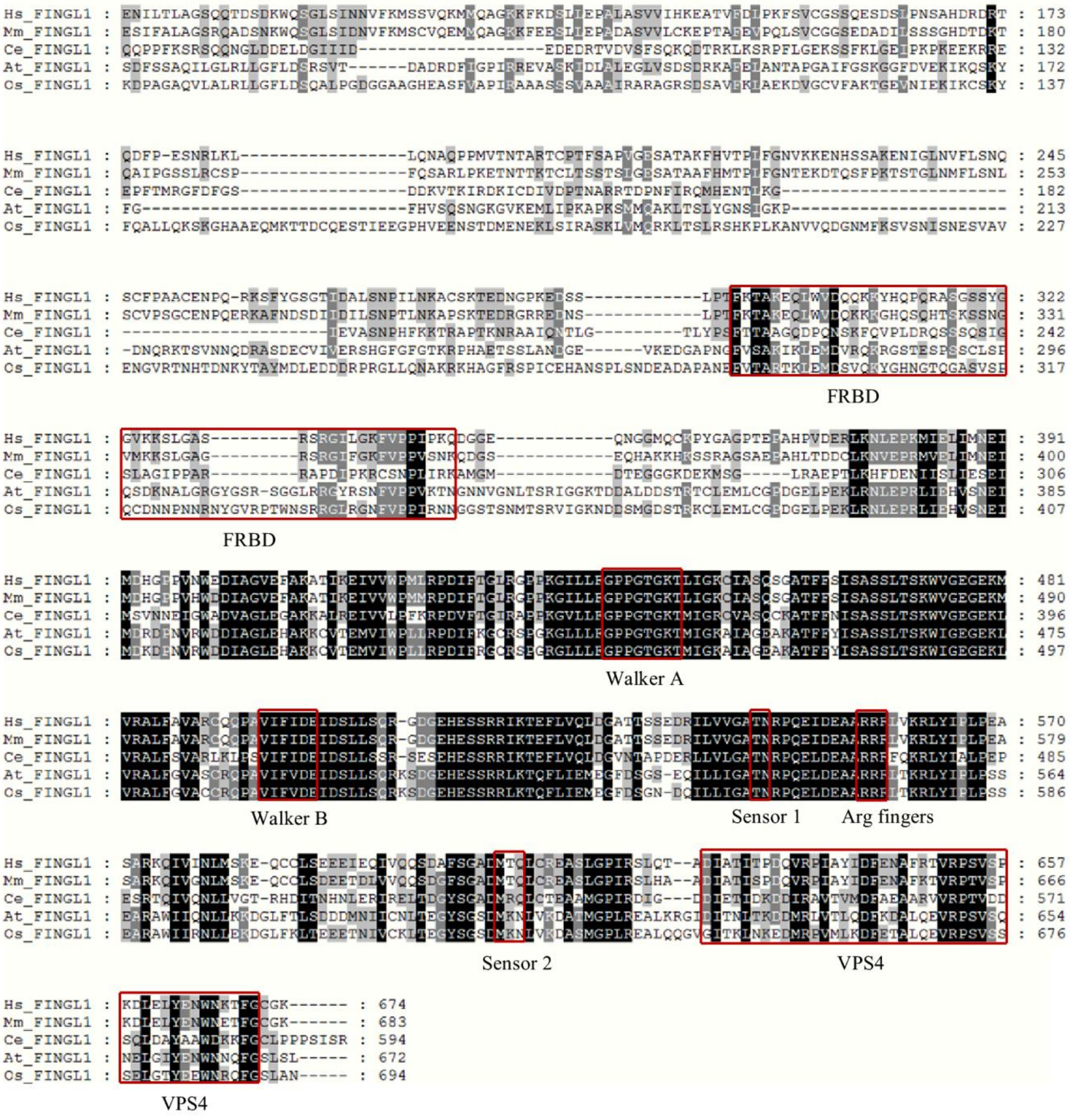

Supplementary Figure S7. Amino acid sequence alignment of OsFIGNL1 from *Homo sapiens*, *Mus musculus*, *Caenorhabditis elegans*, *Arabidopsis thaliana* and *Oryza sativa.* The N-domain was highly divergent between species. Black box represent identical amino acids, and gray boxes highlight similar residue. FRBD domain, Walker A, Walker B, Sensor1, Senson2, Arg fingers and VPS4 are shown in red box. Hs, *Homo sapiens*; Mm, *Mus musculus*; Ce, *Caenorhabditis elegans*; At, *Arabidopsis thaliana*; Os, *Oryza sativa*.

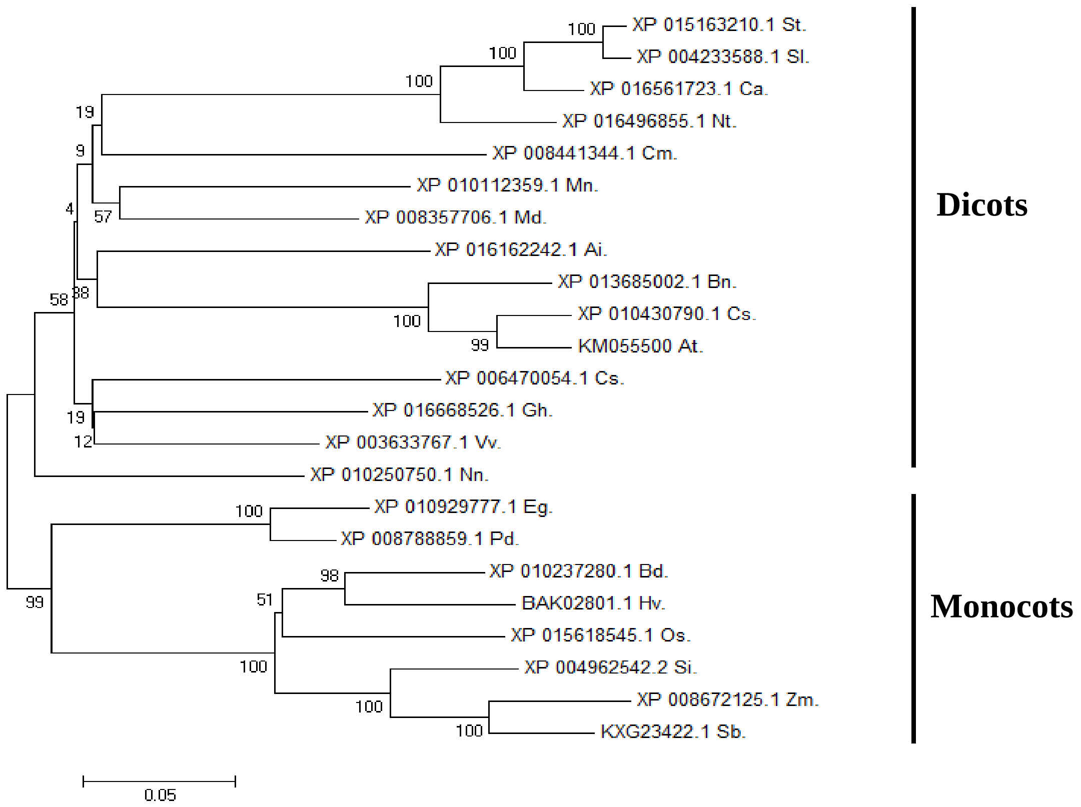

Supplementary Figure S8. Neighbor-Joining phylogenetic tree of OsFIGNL1 family protein. Branches are labeled with GeneBank number. Os, *Oryza sativa*; Bd, *Brachypodium distachyon*; Hv, *Hordeum vulgare*; Si, *Setaria italic*; Sb, *Sorghum bicolor*; Zm, *Zea mays*; Pd, *Phoenix dactylifera*; Eg, *Elaeis guineensis*; Nn, *Nelumbo nucifera*; Md, *Malus domestica*; Vv, *Vitis vinifera*; Ai, *Arachis ipaensis*; Mn, *Morus notabilis*; Gh, *Gossypium hirsutum*; Bn, *Brassica napus*; Cs, *Citrus sinensis*; Cm, *Cucumis melo*; Nt, *Nicotiana tabacum*; Ca, *Capsicum annuum*; St, *Solanum tuberosum*; Sl, *Solanum lycopersicum*; At, *Arabidopsis thaliana*; Cs, *Camelina sativa*.

Supplementary Table S1. Primers used in this study.

Purpose	Name	Forward primer	Reverse primer
Map-based cloning	RM27877	GGAAGCCATGAAAGATGTGTTGC	AATTTCTCCGAGCACCTGAAACG
	InDel162	CCTAGTTCAGCTCCTGCTTACC	GCAGAAGAGAAGTTGTGTGTCG
	S1	GCTCATAATAATGGCCTTGGTTCA	CCTCGGATTTATGTGATGGAGGG
	S2	GGCAAGCTGTATTACTTCTTAACCA	ATGGCTGAACCACCTCATGG
	S3	CTGAAAACTATCCACGCGTCT	CACAGATTTCCAACGGCTGC
	S4	TGCTTTGGTGGTGAGTATGC	ACATTGTGGATAGAAGCACCAC
	S5	CAGACATCGCAGAGCTAGTG	GCCATCTCCGTTGGATGTGT
	S6	TTGGGGTGATCGCTATGTGT	TGTCGATTCTCTGCACCAGC
	S7	CCAGCCTGGGTTTTTGCATC	TGGCTGTCTACATGCAACCC
	S8	GGATGGCAATGCTTCAAGGC	CCTTTTCCCACGTAGTCAGCA
	S9	AGCCGACACTTTGAGCATGA	GAGGAAAATCAGCGGGCAAC
	S10	TGACAAGCCAATGCACAAGC	ACTCTGTGTGACGACGTGAT
	S11	GATGGCAACGGTGCATACAG	TGCGTGGTTAATCTTCCCTT
Primers for gene construction	COM-*OsFIGNL1*	CCATGATTACGAATTCAGTGCTAGCGAGGGTCAGAT	TACCGAGCTCGAATTCCTTCTCACTCACCGTTGCCT
	GFP-*OsFIGNL1*	CGGAGCTAGCTCTAGAAAATGGCGGAGCAGTCTCAC	TGCTCACCATGGATCCATTTGCTAAGCTCCCAAATTGC
CRISPR/Cas9	Cas9-*OsFIGNL1*	AGATGATCCGTGGCAGGGAGGGCCTGCGAGTCGGTTTTAGAGCTATGC	GCATAGCTCTAAAACCGACTCGCAGGCCCTCCCTGCCACGGATCATCT
Identification of transgenic plants	COM-JD	CTGATGGTTTGGATGCGTGTT	TGTGGAATTGTGAGCGGATAA
	Cr-JD	AAATGGCGGAGCAGTCTCAC	CATCCAAATCGCTCCCACTCTT
Y2H	AD-*RAD51A2*	GGAGGCCAGTGAATTCATGTCGTCGTCGGGTGCGG	CGAGCTCGATGGATCCTCAGTCCTTAACATCTGTGACGC
	AD-*RAD51A1*	GGAGGCCAGTGAATTCATGTCGACGTCGGCGGCGG	CGAGCTCGATGGATCCTCAATCCTTGACATCTGCAACG

	AD-*DMC1A*	GGAGGCCAGTGAATTCATGGCGCCGTCCAAGCAGTA	CGAGCTCGATGGATCCTCAGTCTTTCGCATCCATTATTC
	AD-*DMC1B*	GGAGGCCAGTGAATTCATGGCGCCGTCCAAGCAGTAC	CGAGCTCGATGGATCCTCAGTCTTTCGCATCCATTATTC
	BD-*OsFIGNL1*	CATGGAGGCCGAATTCATGGCGGAGCAGTCTCACG	GCAGGTCGACGGATCCTTAATTTGCTAAGCTCCCA
BiFC	*pSPYNE-DMC1A*	CGCCACTAGTGGATCCATGGCGCCGTCCAAGCAGTA	GAGCGGTACCCTCGAGGTCTTTCGCATCCATTATTCCAC
	pSPYNE-DMC1B	CGCCACTAGTGGATCCATGGCGCCGTCCAAGCAGTAC	GAGCGGTACCCTCGAGGTCTTTCGCATCCATTATTCCAC
	pSPYNE-RAD51A1	CGCCACTAGTGGATCCATGTCGACGTCGGCGGCGG	GAGCGGTACCCTCGAGATCCTTGACATCTGCAACGC
	pSPYNE-RAD51A2	CGCCACTAGTGGATCCATGTCGTCGTCGGGTGCGG	GAGCGGTACCCTCGAGGTCCTTAACATCTGTGACGC
	pSPYCE-OsFIGNL1	CGCCACTAGTGGATCCATGGCGGAGCAGTCTCACG	GAGCGGTACCCTCGAGATTTGCTAAGCTCCCAAATTGC
qRT-PCR	S12	AAGTTGGAGATGGATTCTGTGC	ACCCGTGAGGTCATATTGGAAGT
	OsActin1	TGCTATGTACGTCGCCATCCAG	AATGAGTAACCACGCTCCGTCA
